# Supplementary material for: Quantitative analysis of fibroblast migration reveals migratory states characterized by force generation, cell shape and motion
Source: bioRxiv. 2026 May 11:2026.05.06.723282. Preprint. [Version 1] doi: 10.64898/2026.05.06.723282 (PMC13192779; doi:10.64898/2026.05.06.723282)
Supplement: Supplementary file 1 [file NIHPP2026.05.06.723282v1-supplement-1.pdf]

bioRxiv preprint doi: https://doi.org/10.64898/2026.05.06.723282; this version posted May 11, 2026. The copyright holder for this preprint (which was not certified by peer review) is the author/funder, who has granted bioRxiv a license to display the preprint in perpetuity. It is made available under a CC-BY 4.0 International license.

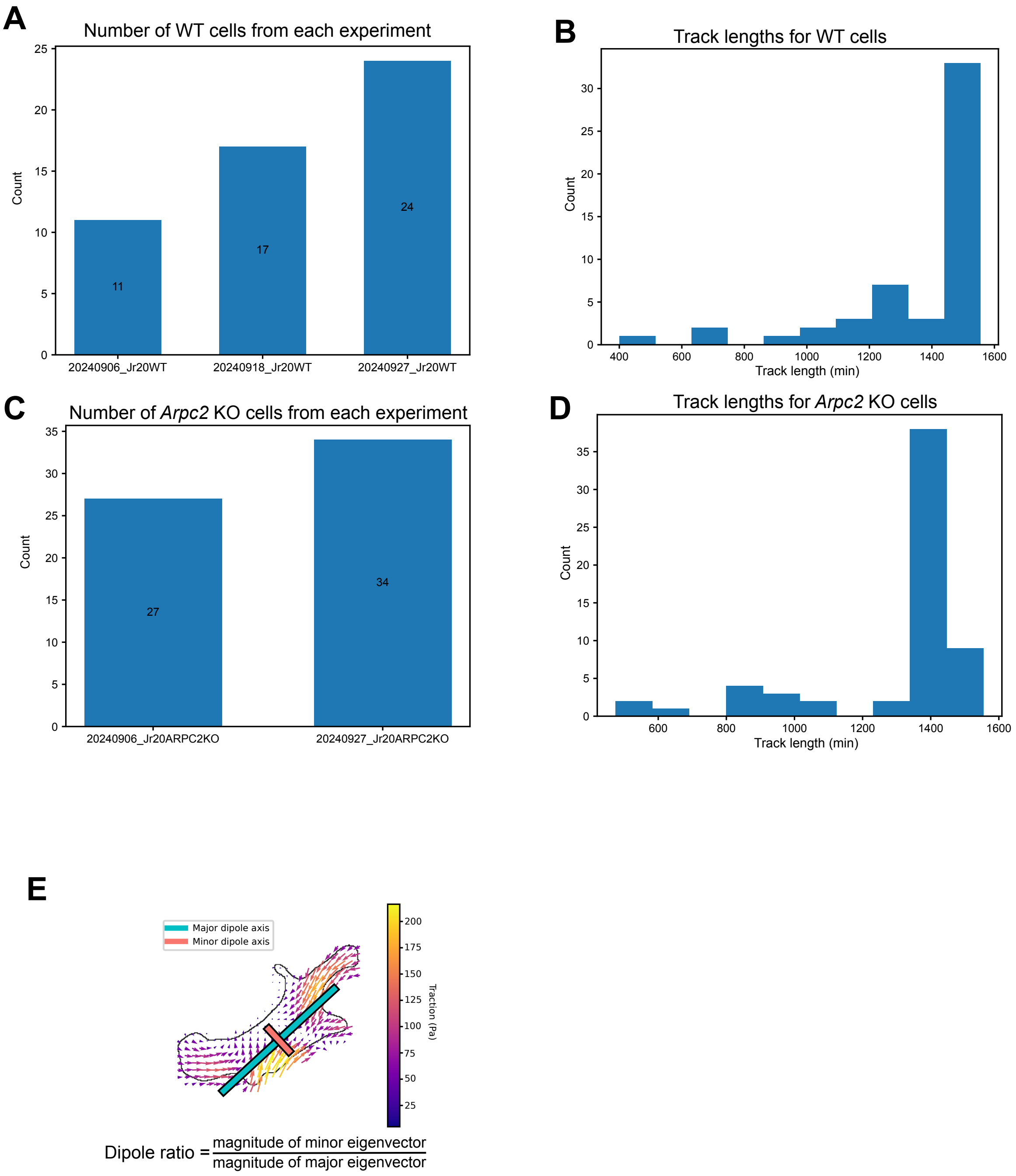

Supplemental Figure 1.
(**A**) Number of WT cells from each independent experiment. (**B**) Histogram showing the track lengths for all the WT cells. (**C**) Number of *Arpc*2 KO cells from each independent experiment. (**D**) Histogram showing the track lengths for all the *Arpc*2 KO cells. (**E**) Diagram displaying the force dipole on an example cell.

bioRxiv preprint doi: https://doi.org/10.64898/2026.05.06.723282; this version posted May 11, 2026. The copyright holder for this preprint (which was not certified by peer review) is the author/funder, who has granted bioRxiv a license to display the preprint in perpetuity. It is made available under a CC-BY 4.0 International license.

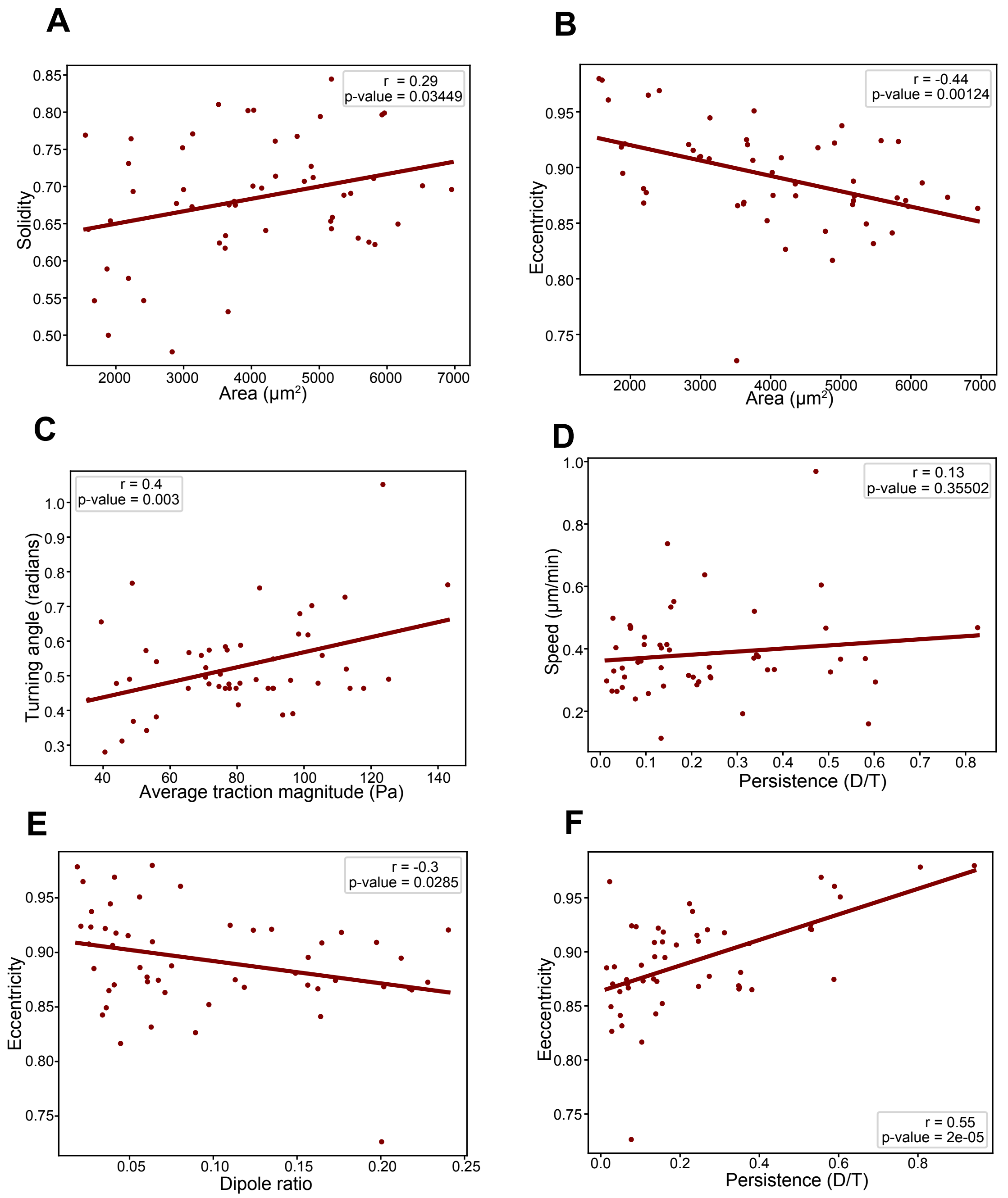

Supplemental Figure 2.

(**A-F**) Scatter plots comparing shape, motility and traction force metrics for WT cells. Each point on the plot represents the median value for one cell over the course of its track (N=52 cells). Linear regression was performed for each plot and the resulting line of best fit is plotted. The Pearson correlation coefficient and associated p-value is displayed on each plot. Statistical significance of the Pearson correlation coefficient is determined with a two-tailed t-test based on the t-distribution with n-2 degrees of freedom. A significance level of 0.05 was used.

bioRxiv preprint doi: https://doi.org/10.64898/2026.05.06.723282; this version posted May 11, 2026. The copyright holder for this preprint (which was not certified by peer review) is the author/funder, who has granted bioRxiv a license to display the preprint in perpetuity. It is made available under a CC-BY 4.0 International license.

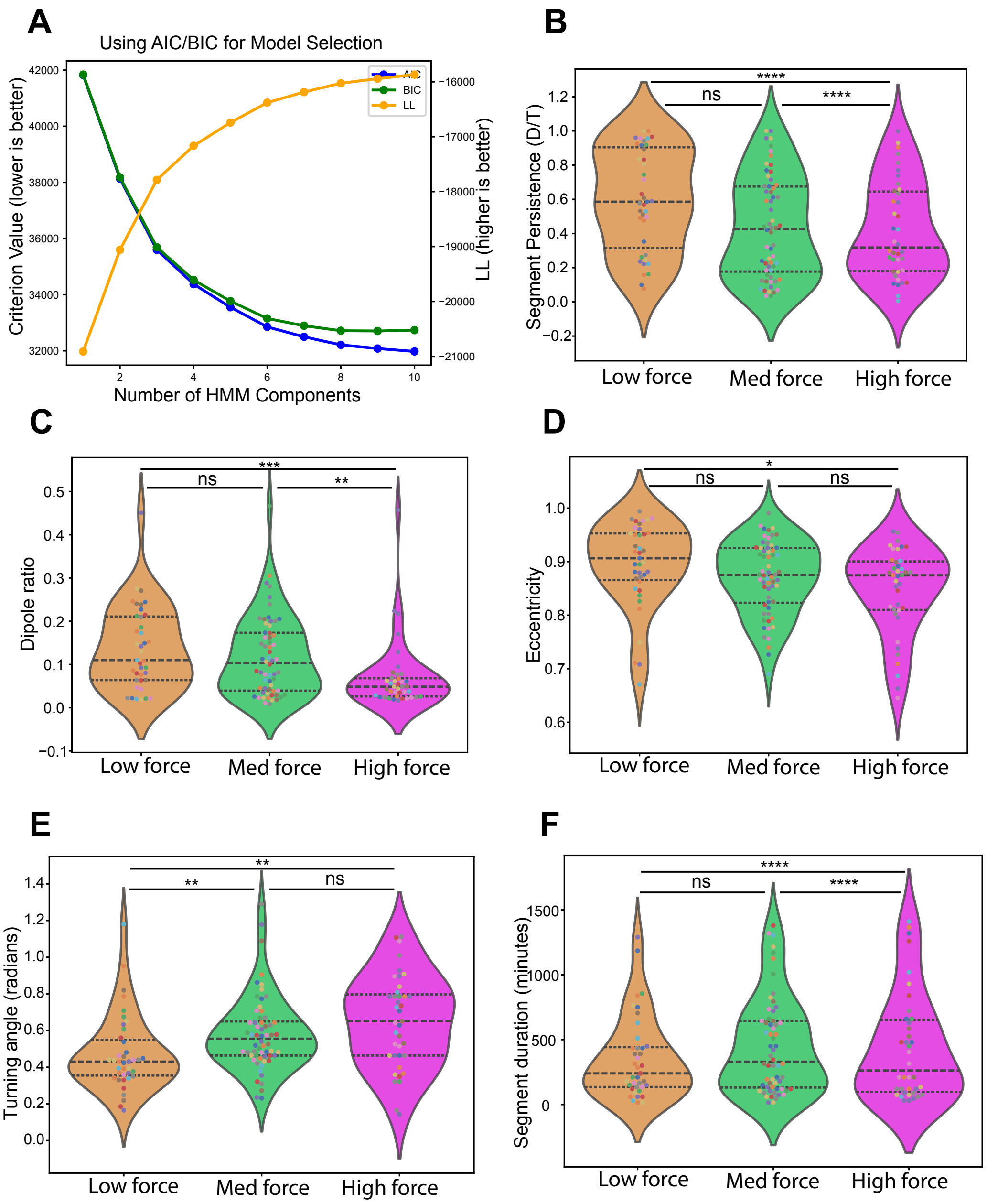

Supplemental Figure 3.
(**A**) AIC (blue), BIC (green), and log-likelihood (yellow) values used to select the appropriate number of states for the Hidden Markov Model for WT cells. (**B-F**) Comparison of median segment values of shape and motion parameters for each HMM state for WT cells. A segment is defined as a series of frames a cell is in one state before it switches states or the track ends. Each dot represents one segment and the colors of the dots correspond to a unique cell (N=52 cells with an average of 2.65 segments per cell). Black dotted lines display the quartiles. Statistical significance is determined with a permutation test comparing the median of the data with the null distribution created from 10,000 permutations with a significance level of 0.05.

bioRxiv preprint doi: https://doi.org/10.64898/2026.05.06.723282; this version posted May 11, 2026. The copyright holder for this preprint (which was not certified by peer review) is the author/funder, who has granted bioRxiv a license to display the preprint in perpetuity. It is made available under a CC-BY 4.0 International license.

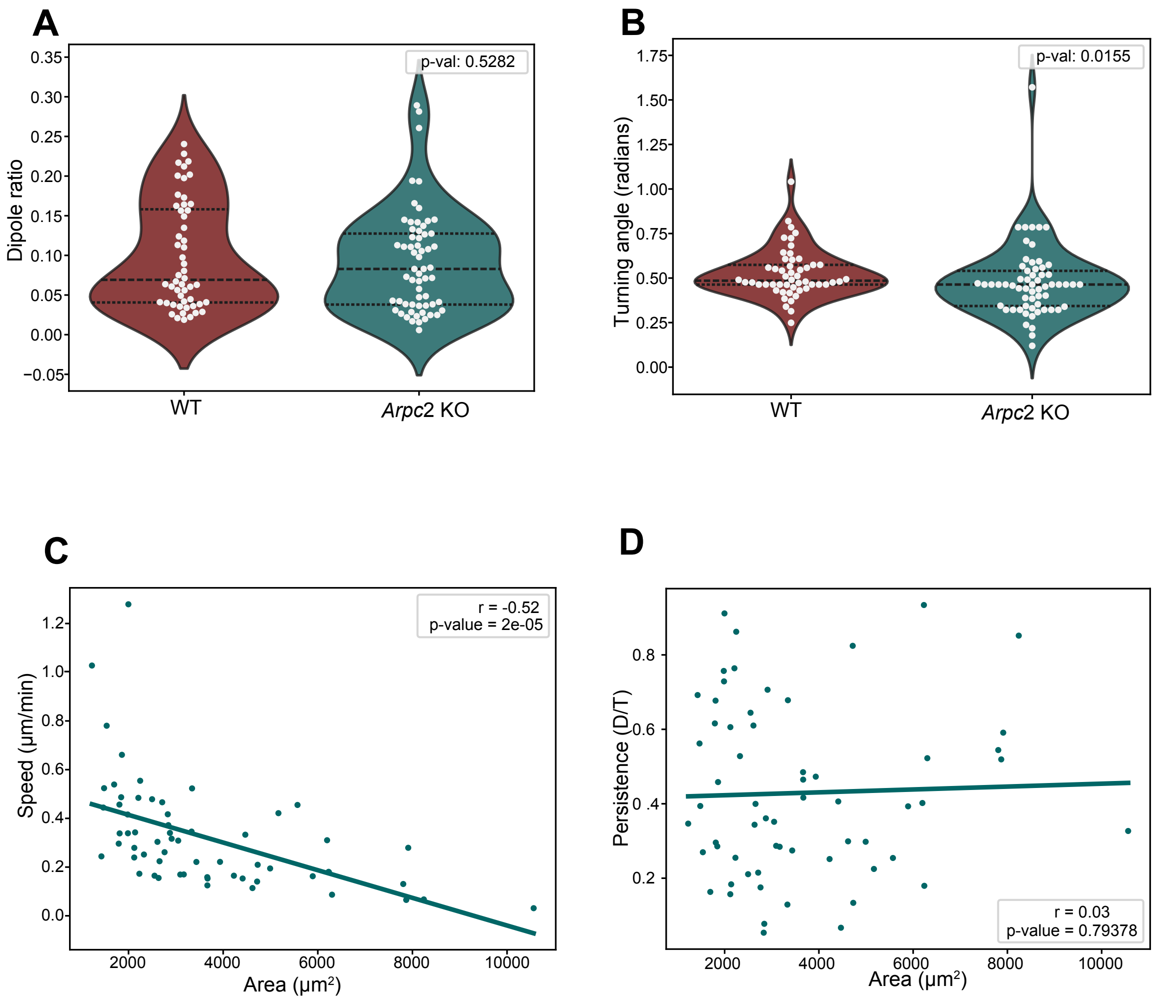

Supplemental Figure 4.
(**A-B**) Comparison of median track values for dipole ratio and turning angle between WT and *Arpc2* KO cells. Each dot represents the median metric value of one cell over the entire course of its track (WT: N=52 cells, *Arpc2* KO: N=61 cells). Black dotted lines display the quartiles. (**C-D**) Scatter plots comparing area and motion for *Arpc2* KO cells. Each point on the plot represents the median value for one cell over the course of its track (N=61 cells). Statistical significance for (**A-B**) is determined with the Mann-Whitney U test. Statistical significance of the Pearson correlation coefficient for (**C-D**) is determined with a two-tailed t-test based on the t-distribution with n-2 degrees of freedom. A significance level of 0.05 was used.

bioRxiv preprint doi: https://doi.org/10.64898/2026.05.06.723282; this version posted May 11, 2026. The copyright holder for this preprint (which was not certified by peer review) is the author/funder, who has granted bioRxiv a license to display the preprint in perpetuity. It is made available under a CC-BY 4.0 International license.

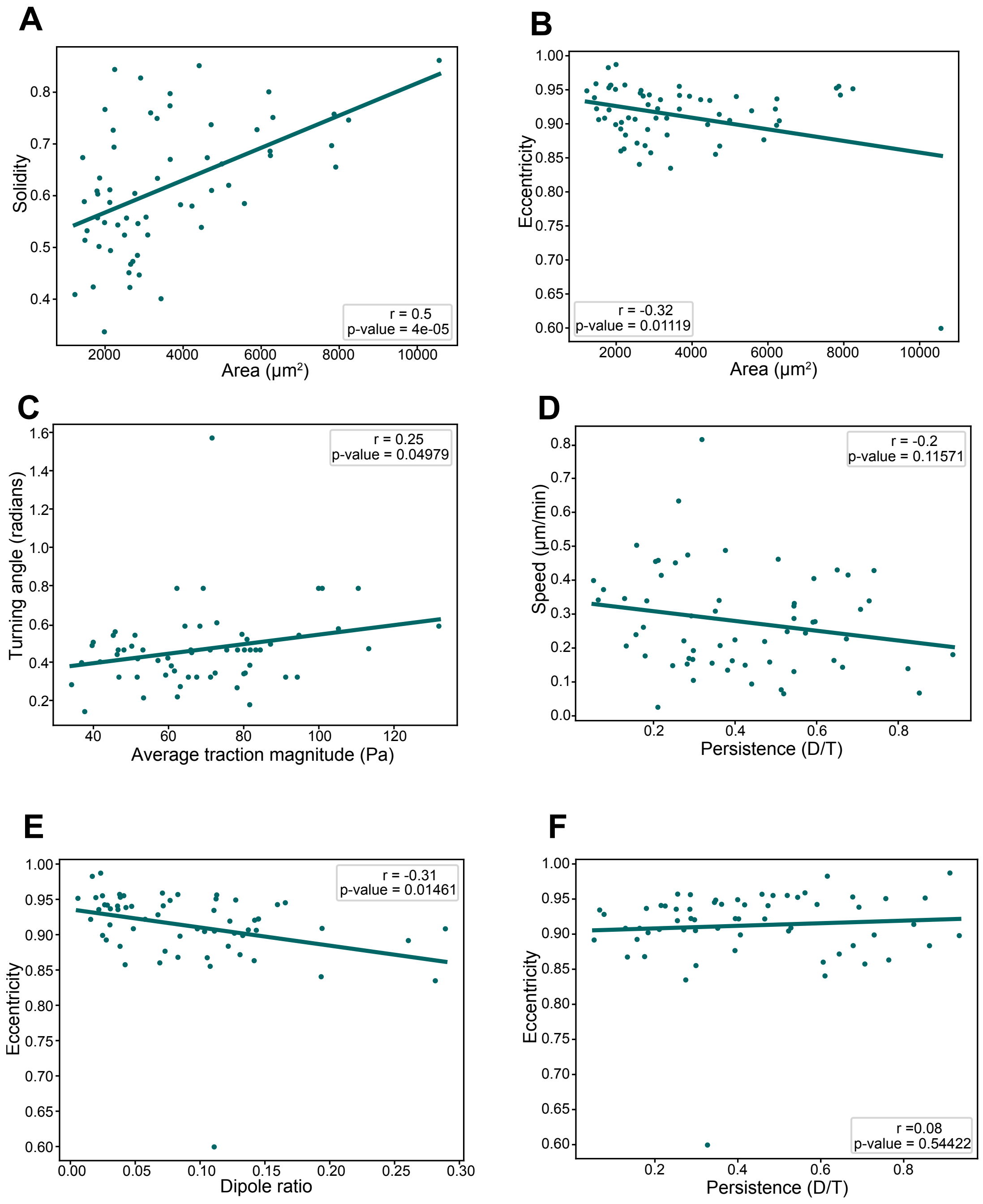

Supplemental Figure 5.
(**A-F**) Scatter plots comparing shape, motion and traction force metrics for *Arpc2* KO cells. Each point on the plot represents the median value for one cell over the course of its track (N=61 cells). Statistical significance of the Pearson correlation coefficient is determined with a two-tailed t-test based on the t-distribution with n-2 degrees of freedom. A significance level of 0.05 was used.

bioRxiv preprint doi: https://doi.org/10.64898/2026.05.06.723282; this version posted May 11, 2026. The copyright holder for this preprint (which was not certified by peer review) is the author/funder, who has granted bioRxiv a license to display the preprint in perpetuity. It is made available under a CC-BY 4.0 International license.

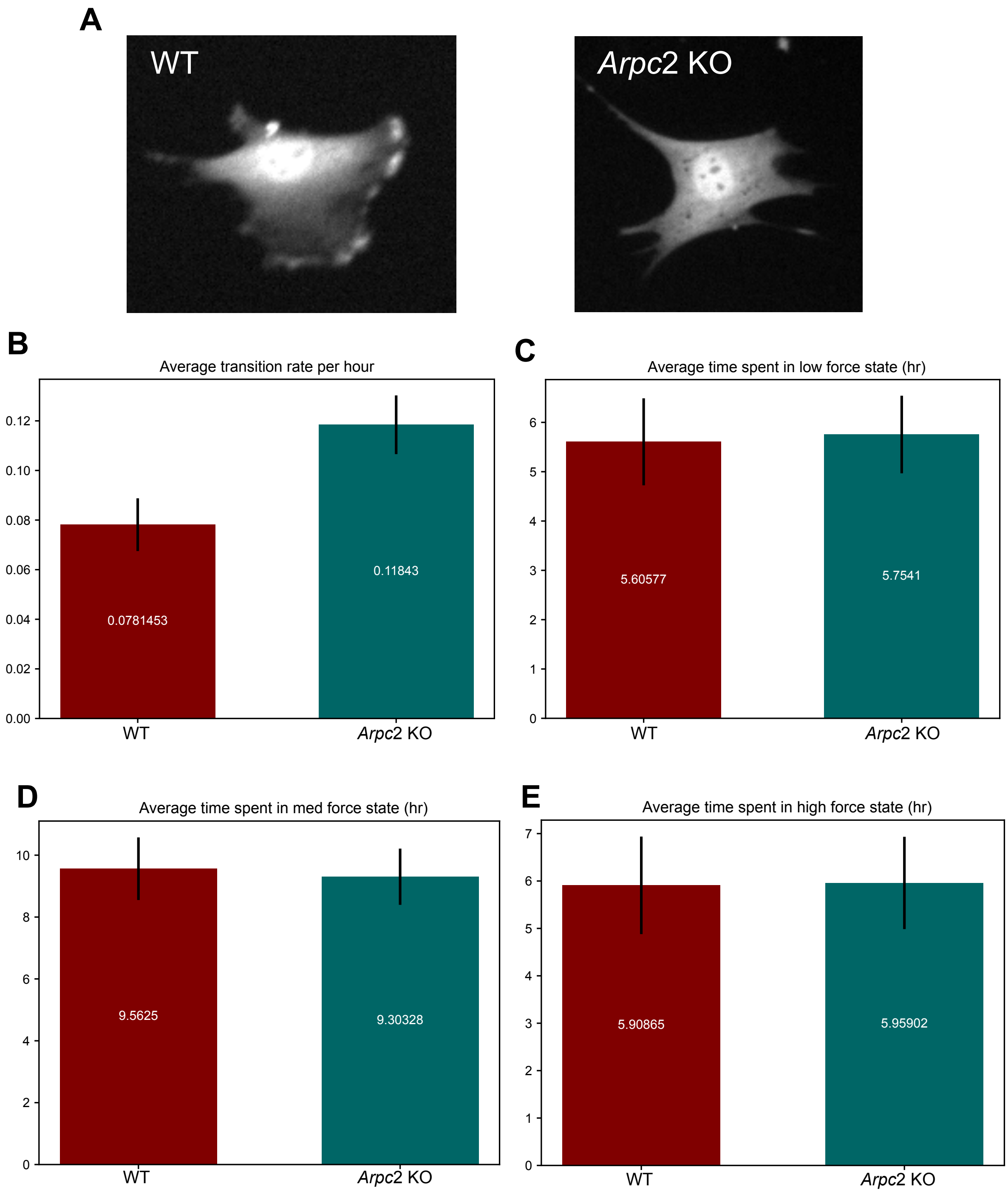

Supplemental Figure 6.
(**A**) Example GFP images of a WT and an *Arpc2* KO cell. (**B**) Average transition rate per hour for WT and *Arpc2* KO cells. (**C-E**) Average time spent in each state in hours. (**B-E**) Means are displayed on the bar with the black lines denoting the standard error of mean (SEM).

bioRxiv preprint doi: https://doi.org/10.64898/2026.05.06.723282; this version posted May 11, 2026. The copyright holder for this preprint (which was not certified by peer review) is the author/funder, who has granted bioRxiv a license to display the preprint in perpetuity. It is made available under a CC-BY 4.0 International license.

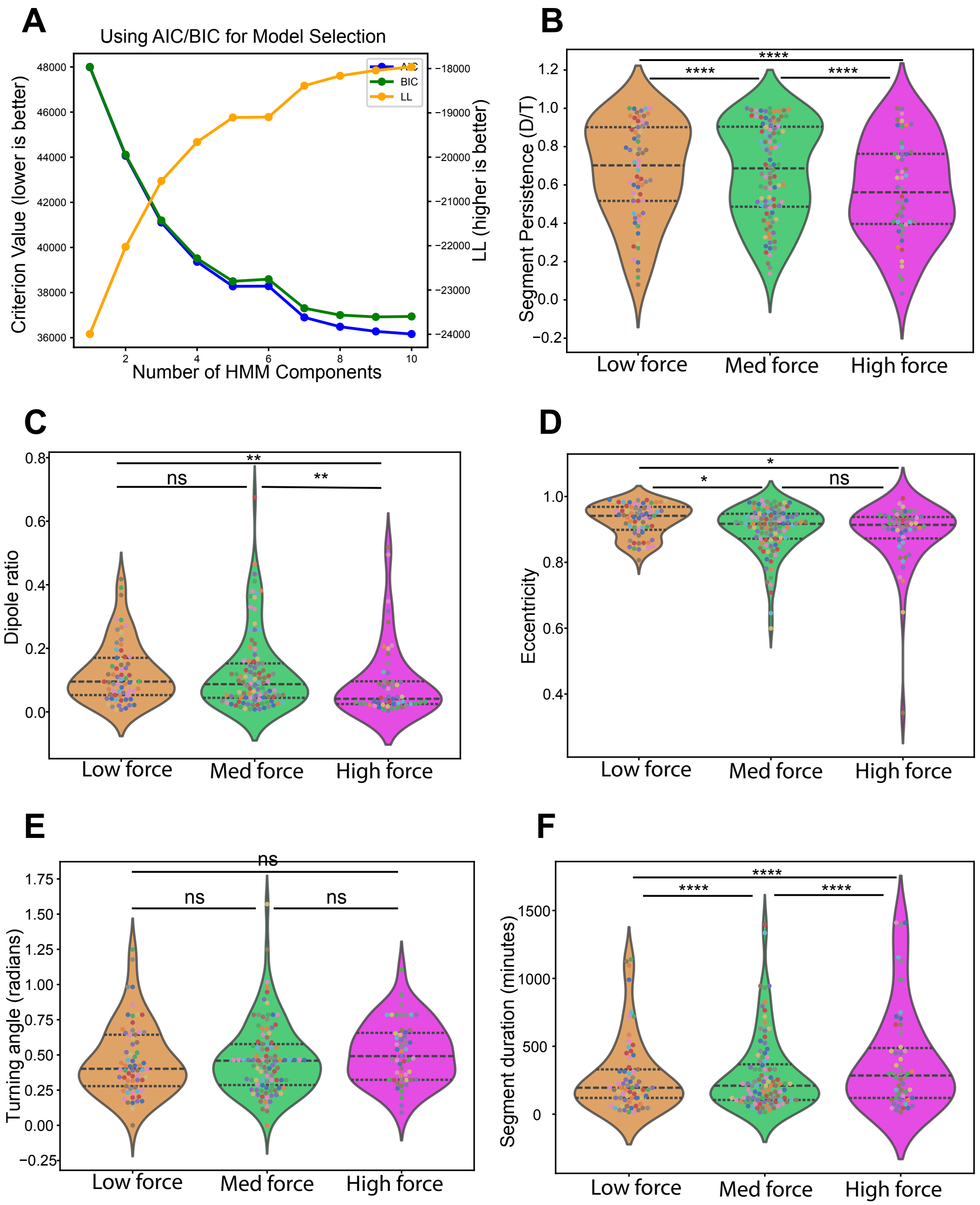

Supplemental Figure 7.
(**A**) AIC (blue), BIC (green), and log-likelihood (yellow) values used to select the appropriate number of states for the Hidden Markov Model for *Arpc*2 KO cells. (**B-F**) Comparison of median segment values of shape and motion parameters for each HMM state for *Arpc*2 KO cells. A segment is defined as a series of frames a cell is in one state before it switches states or the track ends. Each dot represents one segment and the colors of the dots correspond to a unique cell (N=61 cells with an average of 3.49 segments per cell). Black dotted lines display the quartiles. Statistical significance is determined with a permutation test comparing the median of the data with the null distribution created from 10,000 permutations with a significance level of 0.05.

bioRxiv preprint doi: https://doi.org/10.64898/2026.05.06.723282; this version posted May 11, 2026. The copyright holder for this preprint (which was not certified by peer review) is the author/funder, who has granted bioRxiv a license to display the preprint in perpetuity. It is made available under a CC-BY 4.0 International license.

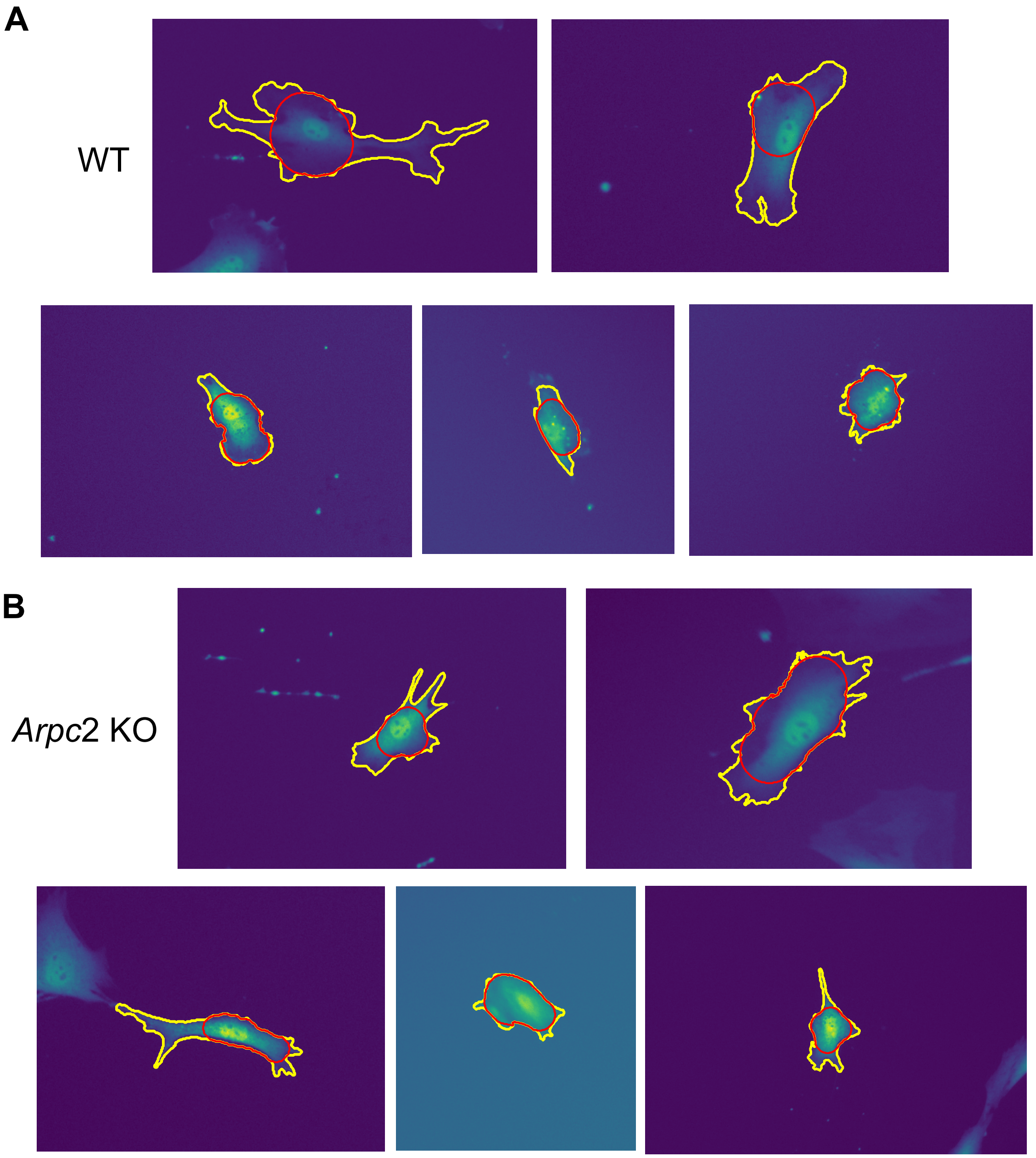

Supplemental Figure 8.
(**A**) WT and (**B**) *Arpc2* KO cells where the cell mask is outlined in yellow and the cell body is outlined in red. The cell body was obtained from morphological opening (Methods). The nucleus is located within the cell body and everything outside the cell body is referred to as a peripheral protrusion.

bioRxiv preprint doi: https://doi.org/10.64898/2026.05.06.723282; this version posted May 11, 2026. The copyright holder for this preprint (which was not certified by peer review) is the author/funder, who has granted bioRxiv a license to display the preprint in perpetuity. It is made available under a CC-BY 4.0 International license.

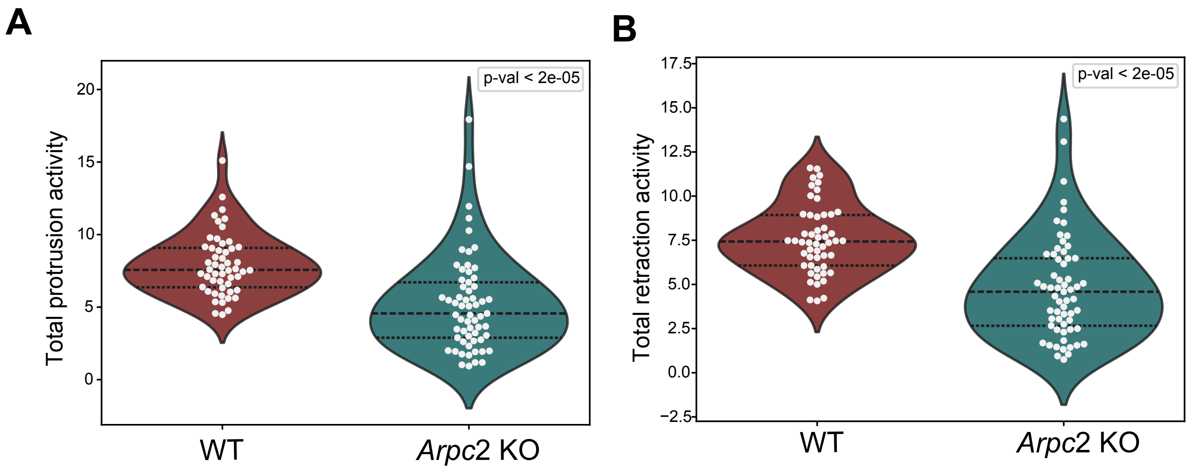

Supplemental Figure 9.
(**A**) Median per cell protrusion boundary activity normalized by cell area and displayed as a percentage. (**B**) Median per cell retraction boundary activity normalized by cell area and displayed as a percentage. WT cells have higher protrusion and retraction boundary activity than *Arpc*2 KO cells. Statistical significance is determined with the Mann-Whitney U test.

bioRxiv preprint doi: https://doi.org/10.64898/2026.05.06.723282; this version posted May 11, 2026. The copyright holder for this preprint (which was not certified by peer review) is the author/funder, who has granted bioRxiv a license to display the preprint in perpetuity. It is made available under a CC-BY 4.0 International license.

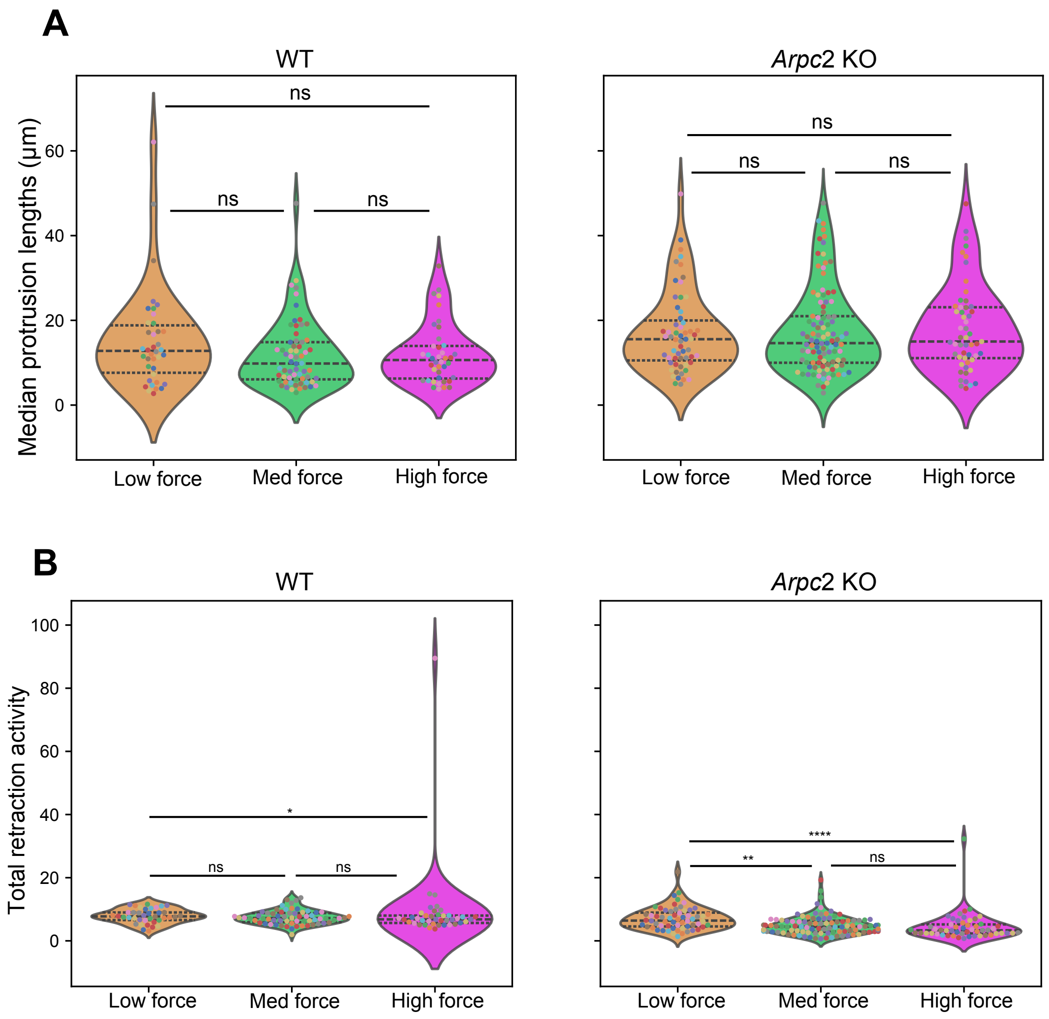

Supplemental Figure 10.
(**A**) The median protrusion length per segment for both WT and *Arpc*2 KO cells in each HMM state. There is no significant difference between the states for either cell type. (**B**) The median retraction activity (normalized by cell area and displayed as a percentage) over a segment within the three predicted hidden Markov model states between WT and *Arpc*2 KO show that the higher force states display slightly lower retraction activity for both WT and *Arpc*2 KO cells. A segment is defined as a series of frames a cell is in one state before it switches states or the track ends. Each dot represents one segment and the colors of the dots correspond to a unique cell. Black dotted lines display the quartiles. Statistical significance is determined with a permutation test comparing the median of the data with the null distribution created from 10,000 permutations with a significance level of 0.05. (WT: N=52 cells, *Arpc*2 KO: N=61 cells)
